# Effect of anti-gliadin IgY antibody on epithelial intestinal integrity and inflammatory response induced by gliadin

**DOI:** 10.1186/s12865-015-0104-1

**Published:** 2015-07-09

**Authors:** Naiyana Gujral, Ju Won Suh, Hoon H. Sunwoo

**Affiliations:** 3142G Katz Group Centre for Pharmacy & Health Research, Faculty of Pharmacy and Pharmaceutical Sciences, University of Alberta, 11361 – 87 Ave, Edmonton, AB T6G 2E1 Canada; Center for Nutraceutical and Pharmaceutical Materials, Myongji University, Cheoin-gu, Yongin, Gyeonggi-Do 449-728 Korea

**Keywords:** Celiac disease, Gliadin, Immunoglobulin Y, Intestinal integrity, Cytokines

## Abstract

**Background:**

Pepsin-trypsin resistant gliadin (PT-gliadin) promotes intestinal tissue inflammation and increases paracellular permeability of immunogenic gliadin peptides into the lamina propria. This leads to the complications seen in the pathogenesis of celiac disease (CD). In this study, specific anti-gliadin IgY antibody was produced and evaluated for its efficacy on gliadin induced intestinal integrity impairment and proinflammatory effects on intestinal epithelial (Caco-2) cell culture model for CD.

**Methods:**

Caco-2 (passages 20-24) monolayers were subjected to 7 experimental conditions (n=3 each): phosphatebufferedsaline (PBS; control), pancreatic digested-casein (PD-casein; negative control), PT-gliadin (positive control), non-specific IgY with PT-gliadin, and anti-wheat gliadin IgY with PT-gliadin at a ratio of 1:6,000, 1:3,000 and 1:1,500. Caco-2 monolayers were then evaluated for effects of gliadin and/or anti-wheat gliadin IgY after 24 h exposure. Enzyme-linked immunosorbent assay (ELISA) was used to quantify anti-inflammatory markers (TNF-α and IL-1β) 5 days after cells were exposed to PT-gliadin and/or anti-wheat gliadin IgY.

**Results:**

Among other conditions, anti-wheat gliadin IgY at a ratio of 1:3,000 (anti-gliadin IgY: PT-gliadin) significantlyprevented gliadin toxicity on Caco-2 by maintaining intestinal integrity, inhibiting phenol red permeation, and inhibiting gliadin absorption and production of proinflammatory cytokines (TNF-α and IL-1β) as compared to PT-gliadin stimulated cultures (*P* < 0.05).

**Conclusion:**

The anti-wheat gliadin IgY antibody produced in this study has proved to inhibit absorption of gliadin and gliadin-induced inflammatory response in Caco2 cell culture model of CD. Anti-gliadin IgY, therefore has potential to be used as an oral passive antibody therapy to treat CD.

## Background

Celiac disease is one of the most common autoimmune diseases, occurring in 1 out of 100–300 people worldwide [1]. CD is driven by an abnormal immune response to the ingestion of gluten in genetically (HLA DQ2/DQ8) predisposed individuals. Among these gluten proteins, gliadin is exceptionally resistant to enzymatic degradation due to its high proline and glutamine content [2]. The PT-gliadin can cross the membrane of enterocytes and provoke damage in a variety of ways. Firstly, the binding of gliadin to the CXCR3 receptor results in the increased release of the protein zonulin, which can lead to impaired mucosal integrity. Gliadin can then enter enterocytes by transcytosis or retrotranscytosis via secretory IgA through the transferrin receptor CD71 [3]. The release of p31–43/49 peptides triggers the innate immune response [4], localizes to endocytic vesicles which leads to the production of inflammatory cytokines, such as IL-1β and TNF-α, and impaired mucosal integrity [5]. P31-43 interferes with the endocytic pathway by causing delay maturation of early endosomes to late endosomes, thus affecting various metabolic pathways and cellular functions [6, 7]. The 33-mer (p56-89) gliadin can also penetrate into the lamina propria, triggering the T-helper cell mediated adaptive immune response [8] that contributes to the ongoing inflammation in the small intestine of CD patients [9].

Currently, there is no pharmacological therapy available for CD patients. Only strict adherence to a GFD helps to alleviate symptoms. Finding a more convenient, safe, and cost-effective way of relieving symptoms would contribute greatly to the quality of life for these patients, and one avenue of study includes how to prevent triggering the immune system upon the ingestion of gluten. Potential therapeutic options include the hydrolysis of toxic gliadin by exogenous enzymes [10], the modification of gliadin-derived peptide pattern by Bifidobacteria [8], the prevention of gliadin absorption by polymeric binders [11], the inhibition of tight junction opening by zonulin antagonists [12], the blockage of selective deamidation of specific glutamine residues by tissue tranglutaminase inhibitors [13], the restoration of immune tolerance towards gluten by vaccines [14], the modulation of immune response to gliadin by NKG2D/MICA blockers [15] and the neutralization of gliadin *in vivo* by IgY antibody [16].

Among these, oral passive immunotherapy may be the best avenue to pursue simply by virtue of its advantages, including safety, reduced cost, ease of administration, and potential to treat localized conditions in the gastrointestinal tract (GIT) [17]. Chicken egg yolk immunoglobulin (IgY) is ideal for passive immunotherapy, as it may be readily obtained in large quantities from egg yolk. Compared to the traditional method of harvesting mammalian antibodies, IgY purification is more cost-effective, convenient, and hygienic. IgY antibodies have already been shown to be effective in neutralizing disease-causing pathogens such as *Rotavirus* [18], *E. coli O157:H7* [19], *Salmonella enteritis* [20], *Clostridium perfringens* [21]. Despite this, there is only limited information available that describes the use of IgY antibodies in neutralizing toxic gliadin in an intestinal epithelium culture system. The Caco-2 cell line has been used in several studies as an *ex vivo* model of CD intestinal epithelia for initial testing of novel CD treatment options [22–24]. In this study, Caco-2 cell cultures were used to evaluate the effectiveness of anti-gliadin IgY in inhibiting gliadin induced impaired intestinal integrity, gliadin absorption and the inflammatory response induced by gliadin.

The objectives of this study are to produce anti-gliadin IgY antibodies by immunizing chickens with gliadin, to purify the resultant IgY antibodies by gel chromatography, and to characterize its reactivity to gliadin by western blot and ELISA techniques. The anti-gliadin IgY antibodies were then tested for its efficacy in preventing gliadin induced impaired intestinal integrity and inflammatory response in Caco-2 cell cultures.

## Results

### Production of anti-gliadin IgY

**Fig. 1:**
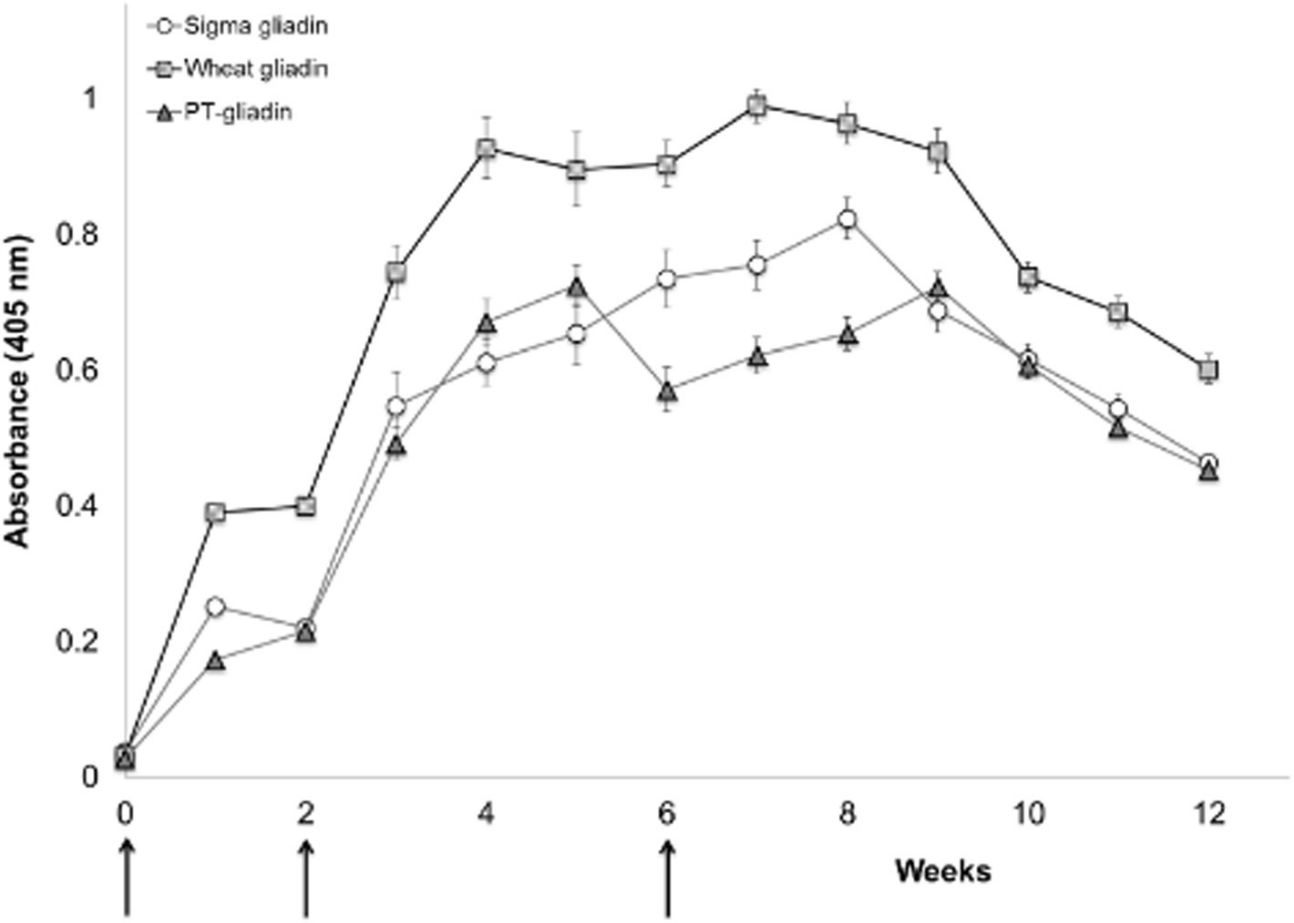
Specific IgY antibody ELISA values in the egg yolk from chickens immunized with Sigma gliadin (SG), wheat gliadin (WG), and pepsin-trypsin resistant gliadin (PT-gliadin) (500 μg/ml protein) in PBS, emulsified with Freund’s incomplete adjuvant. Booster immunizations were given at 2 and 6 weeks after the initial immunization. Values are the mean of quadruple samples, with vertical bars indicating the standard deviation. Arrows indicated times of immunization

The anti-gliadin IgY antibodies obtained from chickens immunized with Sigma gliadin, wheat gliadin or PT-gliadin were weekly titrated by indirect ELISA. As shown in Fig. 1, the titre of anti-gliadin IgY was undetectable on day 0, rapidly increased (P > 0.05) from week 2 to 4, and then remained relatively constant (P > 0.05) during week 5–7 periods. Among three gliadins, chickens produced the highest anti-gliadin IgY in response to wheat gliadin (P > 0.05), throughout the immunization period. Therefore, anti-wheat gliadin IgY was used for further studies.

**Fig. 2:**
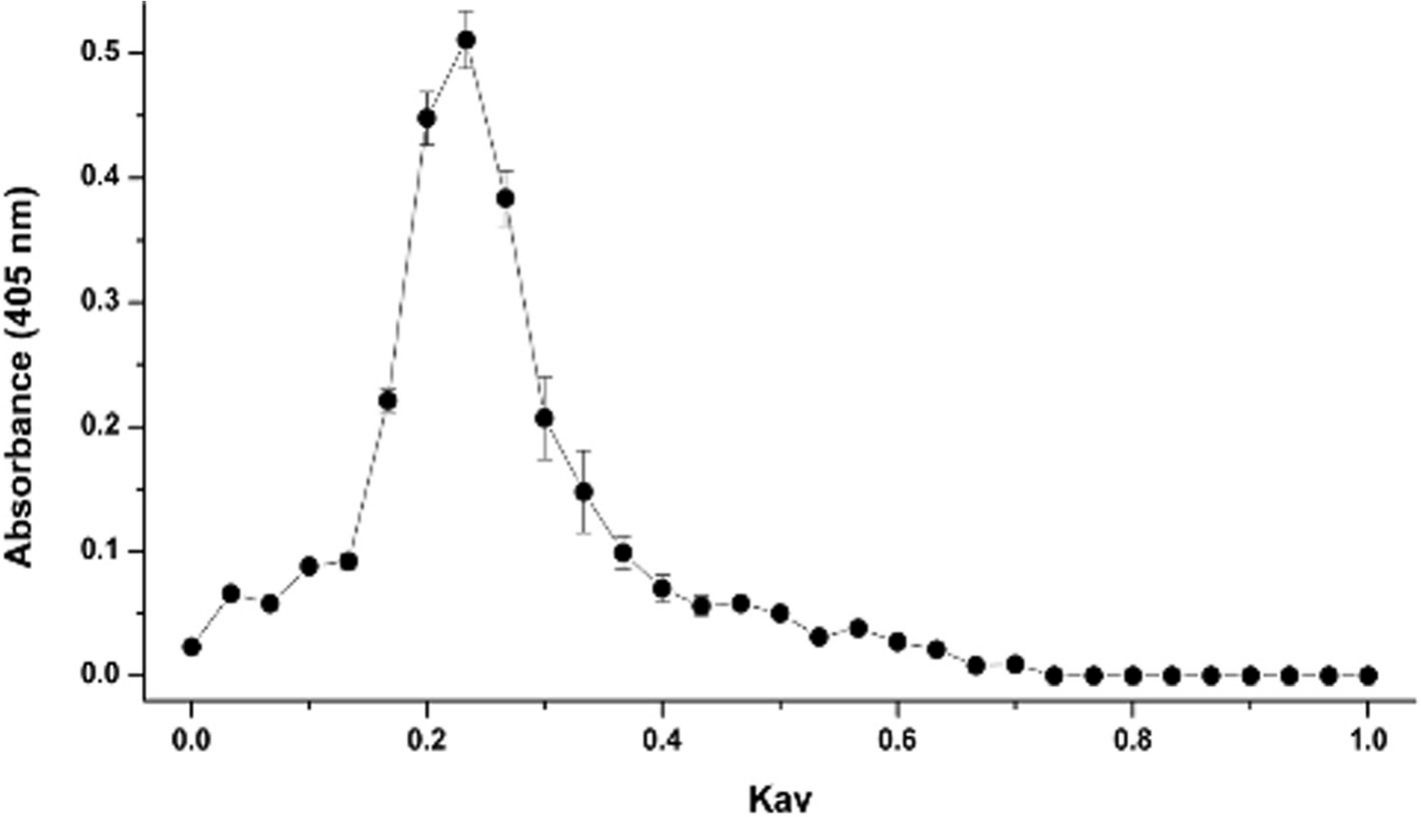
IgY purification by Sephacryl S-300 chromatography. Each value of absorbance was determined by an indirect ELISA. Flow rate: 3 ml/h, Column: 1.0 × 110 cm

After 5–7 weeks of immunization, the egg yolks from wheat gliadin immunized chickens were pooled for purification of IgY by Sephacryl S-300 gel chromatography. The elution profile shows that fractions corresponding to K_av_ 0.2–0.24 contain IgY determined by indirect ELISA (Fig. 2). Yield of total IgY as shown in Table 1 was similar regardless of the different gliadin immunizations (P > 0.05). However, the concentration of specific anti-gliadin IgY was significantly higher in Sigma gliadin, wheat gliadin, and PT-gliadin immunized chickens than in the non-immunized chickens (*P* < 0.05). Sephacryl S-300 purified IgY fractions against Sigma gliadin, wheat gliadin, and PT-gliadin contain approximately 7.9 %, 8.1 %, and 7.7 % of specific anti-gliadin IgY in a total of IgY antibodies, respectively.

**Table 1:** Concentrations of protein, total IgY, and specific IgY in purified IgY solution by Sephacryl S-300 chromatography

IgY	Concentration (μg/ml)			Specific IgY/Total IgY (%)
	Protein	Total IgY	Specific IgY	
Anti-Sigma gliadin	150.1 ± 1.5	136.4 ± 2.9	10.77 ± 0.05	7.9 %
Anti-wheat gliadin	152.0 ± 1.4	136.9 ± 3.3	11.05 ± 0.09	8.1 %
Anti-PT gliadin	149.0 ± 1.2	135.8 ± 3.0	10.41 ± 0.07	7.7 %
Non-specific	140.5 ± 1.7	127.5 ± 4.4	0.79 ± 0.005	0.01 %

Values are the mean of quadruple samples ± SD

**Fig. 3:**
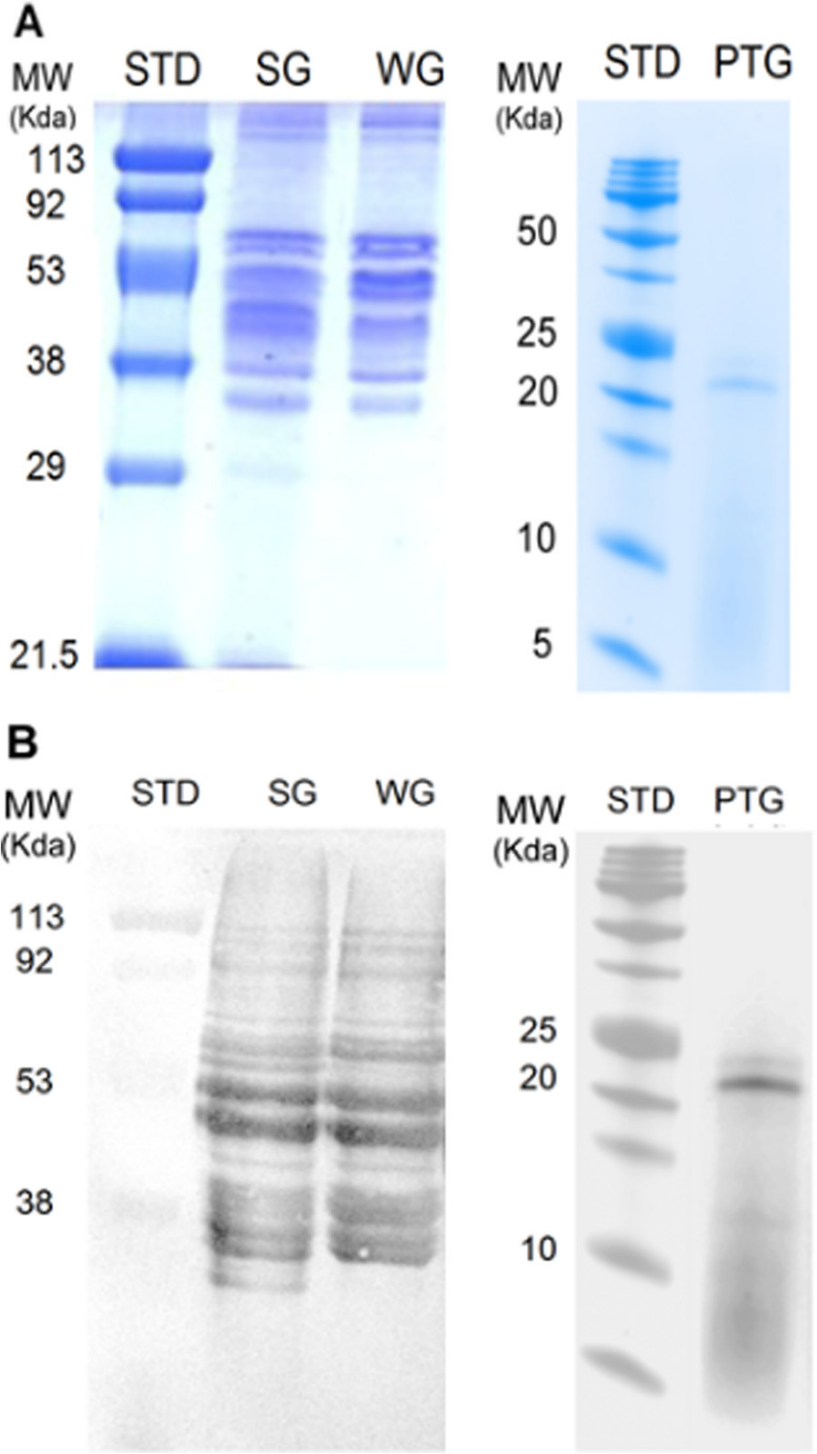
Gliadin fractions from Sigma: SG; Wheat: WG; and; pepsin-trypsin digested Sigma-gliadin: PTG (10 μg/well protein) on **a**) Electrophoresis gel stained with Comassie blue and; **b**) Western blot assay with anti-wheat gliadin IgY

Sigma and wheat gliadins showed similar molecular weight in the range of 30–70 kDa analyzed by SDS-Urea-PAGE (Fig. 3a). On the other hand, the SDS-PAGE showed that PT-gliadin contained a single peptide band at 22 kDa. Western blot assay (Fig. 3b) was performed to determine the anti-wheat gliadin IgY reaction to the three gliadins. The result shows that the anti-wheat gliadin IgY reacted with Sigma and wheat gliadins at a range of 30–70 kDa and PT-gliadin at 22 kDa, indicating the cross-reactivity of the anti-wheat gliadin IgY to other gliadin fragments separated by the electrophoresis. Another method, ELISA, was used to determine the cross-reactivity of the anti-wheat gliadin IgY to Sigma gliadin, PT-gliadin, barley prolamin, rye prolamins, oat prolamins and non-prolamin containing grains (rice and corn). The cross reactivity was calculated based on reference to 100 % reactivity to wheat gliadin (OD_405_ = 1.152). The cross-reactivity of anti-wheat gliadin IgY was highest to Sigma gliadin (99.3 %), followed by barley prolamin (91.3 %), rye prolamin (80.2 %) and oat prolamin (42.7 %). Anti-wheat gliadin IgY also showed high cross-reactivity to PT-gliadin (98 %), whereas the antibody cross-reactivity for non-prolamin-containing grains was neglectable (<1 %). Non-specific IgY, used as negative control antibody, showed no cross reactivity to the prolamin-containing grains mentioned above.

### IgY neutralization of toxic gliadin

**Fig. 4:**
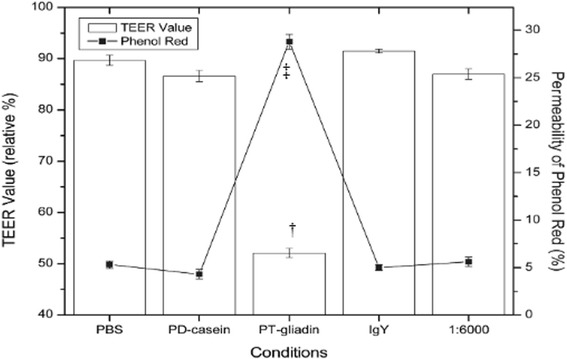
Relative TEER and phenol red permeation measurements to determine effects of anti- gliadin IgY in Caco-2 cells. Pancreatic digested casein (PD-casein) (60 µg); PT-gliadin (60 µg); Non-specific IgY (40 ng); Anti-gliadin IgY (10 ng) and PT-gliadin (60 µg) [1:6,000]. † indicates statistically significant decrease in TEER value (P < 0.05). ‡ indicates statistically significant permeation of phenol red (*P* < 0.05). Values are shown as mean ± SD. Analysis of each group was done in triplicates per plate. Five plates were repeated (*n* = 15)

To evaluate the neutralizing effect of anti-gliadin IgY on gliadin-induced intestinal integrity deterioration, Caco-2 cells were exposed to different ratios of anti-gliadin IgY and PT-gliadin. At 21 days, after seeding cells on the transwell inserts, the TEER values were in the range of 305–310 Ω cm^2^ in well-formed Caco-2 monolayers. Caco-2 monolayers have non-significant changes in TEER values after 4 h in PBS, PD-casein and non-specific IgY (control conditions) (P > 0.05). Upon 4 h exposure to PT-gliadin there was a significant decrease in TEER value of 52 %, as compared to exposures to negative control conditions in TEER value of 85 %. Basal TEER value of Caco2 monolayers at time zero is considered to have TEER value of 100 %. When the Caco2 monolayers were exposed to anti-gliadin IgY and PT-gliadin at a ratio of 1: 6,000 [anti-gliadin IgY (10 ng) and PT-gliadin (60 μg)] for 4 h, (P > 0.05) (Fig. 4), there was no significant TEER value change. This indicates that anti-gliadin IgY neutralized the toxic gliadin and prevented gliadin-induced impairment of intestinal integrity. Anti-gliadin IgY at higher ratio (1:3,000 and 1:1,500) showed similar effects to Caco2 monolayers exposed to anti-gliadin IgY and PT-gliadin at a ratio of 1: 6,000 (data not shown).

The monolayer integrity was determined TEER in the range of 305–310 Ω cm^2^ which is mirrored by < 1 % passage of phenol red from the apical to basal chamber. Phenol red was added to the apical chamber to determine its permeability into the basal chamber after 4 h of exposure. Upon 4 h incubation with PBS and PD-casein as controls, < 6 % phenol red permeated into the basal chamber and < 10 % reduction in TEER value (Fig. 4), indicating minimal disturbed intestinal integrity. After incubation for 4 h with PT-gliadin, there was a significant permeation of 28.8 % phenol red into the basal chamber (compared to negative controls, < 6 %) and relative TEER value of approximately 52 % (compared to negative control, 85 %) (*P <* 0.05). Incubation with anti-gliadin IgY alone did not affect the intestinal integrity. When the monolayers were exposed to three different ratios of anti-gliadin IgY and PT-gliadin for 4 h, there was minimal phenol red permeation of 5.0-5.6 % (P > 0.05), indicating that anti-gliadin IgY successfully prevented gliadin induced impairment of intestinal integrity at a ratio of 1:6,000.

### Effect of anti-gliadin IgY on gliadin permeation in Caco2 monolayer

PT-gliadin was added to the apical chamber with/without anti-gliadin IgY to determine its permeability from the apical chamber into the basal chamber. Upon Caco-2 monolayer exposure to PT-gliadin for 4 h, significant decrease in Caco-2 monolayer integrity was observed, resulting in PT-gliadin translocation of 14.8 % of total PT-gliadin subjected to the apical chamber into the basal chamber (*P* < 0.05). This is contrasted with the exposure to three different ratios of anti-gliadin IgY and PT-gliadin for 4 h, where the passage of gliadin into the basal chamber was inhibited leading to undetectable levels of PT-gliadin in the basal chamber. Table 2 showed that approximately 94 % of PT-gliadin was recovered in the apical chambers of monolayers exposed to three different ratios of anti-gliadin IgY and PT-gliadin for 4 h. However, the PT-gliadin was not detected in basal chamber, indicating that the rest of gliadin (approximately 6 %) may be located in the epithelial cells. Thus, anti-gliadin IgY neutralized PT-gliadin in the apical chamber, blocking gliadin absorption through the Caco-2 monolayer.

**Table 2:** Gliadin content quantified in the apical and basal chamber of Caco-2 cell cultures exposed to digested gliadin with/without anti-wheat gliadin

Condition	% Gliadin					
	Apical			Basal		
	1 h	2 h	4 h	1 h	2 h	4 h
A	ND	ND	ND	ND	ND	ND
C	98 ± 3	90.3 ± 3	76.3 ± 3*	ND	2.5 ± 0.5	14.8 ± 1**
D	ND	ND	ND	ND	ND	ND
E	96.2 ± 1	93.5 ± 1	94.2 ± 1	ND	ND	ND
F	94.0 ± 1	93.7 ± 1	94.9 ± 1	ND	ND	ND
G	97 ± 1	94.9 ± 1	94.5 ± 1	ND	ND	ND

A: Phosphate buffered saline (PBS)

C: Pepsin-trypsin digested gliadin (PT-gliadin)

D: anti-wheat gliadin

E: PT-gliadin: anti-gliadin IgY (1:6,000)

F: PT-gliadin: anti-gliadin IgY (1:3,000)

G: PT-gliadin: anti-gliadin IgY (1:1,500)

Data from a representative experiment performed are shown as mean ± SD (*n* = 15)

* indicate statistically significant lower gliadin content in apical chamber (*P < 0.05*)

** indicate statistically significant higher gliadin content in basal chamber (*P < 0.05*)

ND: Not detectable

### Quantification of inflammatory markers

**Fig. 5:**
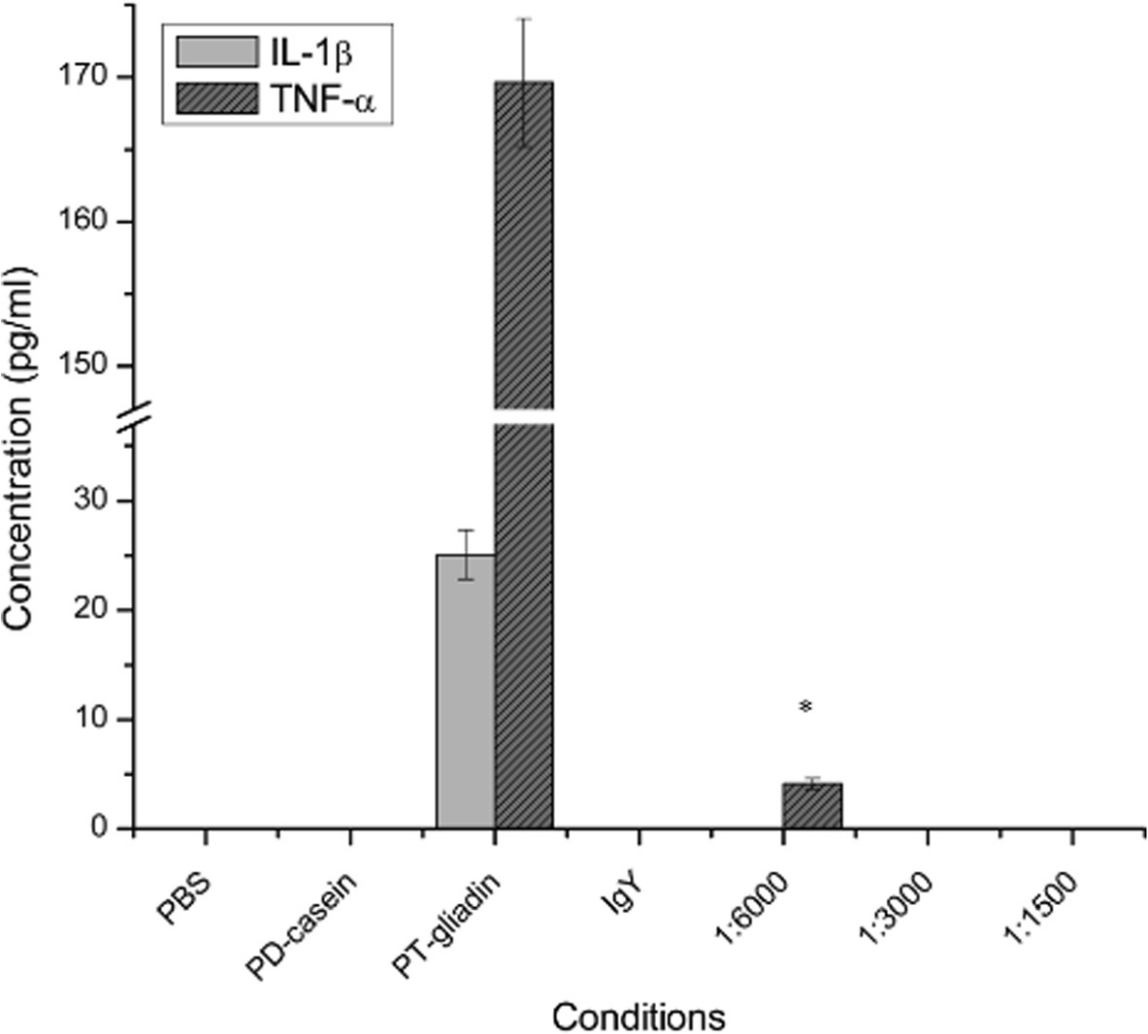
Pro-inflammatory cytokine (IL-1β and TNF-α) in Caco-2 cell. Results are expressed as mean ± SD (*n* = 9). * indicates statistically significant decrease in TNF-α production (*P* < 0.05)

Figure 5 illustrates that PT-gliadin stimulated the synthesis of cytokines, IL-1β and TNF-α in Caco-2 cells after 24 h incubation. Upon PT-gliadin stimulation with PT-gliadin incubation with anti-gliadin IgY at a ratio of 1:6,000 [anti-gliadin IgY (10 ng) and PT-gliadin (60 μg)], a 6.77 folds higher of TNF-α content than that of IL-1β was detected in the Caco-2 cell culture supernatant. IL-1β and TNF-α concentration in the cell supernatant were significantly decreased (*P* < 0.05). However, other two combinations with higher content of anti-gliadin IgY of 1: 3,000 [anti-gliadin IgY (20 ng) and PT-gliadin (60 μg)], and 1,500 [anti-gliadin IgY (40 ng) and PT-gliadin (60 μg)], showed undetectable levels of TNF-α. On the other hand, IL-1β levels remained undetectable with all three ratios of anti-gliadin IgY co-incubations with PT-gliadin. No cytokines were detected in cultures exposed to control PBS, PD-casein, and non-specific IgY.

## Discussion

CD is caused by the exposure of PT-gliadin containing toxic 13-mer (p31-43) and 33-mer (p56-88) peptides [25–27], to the intestinal lumen of genetically susceptible individuals. The PT-gliadin is absorbed from intestinal lumen into the gut mucosa through transcellular and paracellular means [28], causing the pathophysiologic processes in CD and its clinical presentations.

Several therapeutic approaches have been attempted to neutralize or prevent gliadin absorption. As this is the initial step of gliadin-induced toxicity in CD individuals, it would prevent further damage to enterocytes. ALV003, a mixture of glutenase and endoprotease, was reported to enzymatically hydrolyze toxic gliadin peptides [10]. These enzymes may hydrolyze peptides other than gliadin in the GIT. Another candidate being extensively studied is Larazotide, which inhibits zonulin, and in turn prevents toxic gliadin peptide absorption [12]. This drug candidate inhibits the paracellular route of gliadin absorption through tight junctions, but would not fully inhibit gliadin-induced damage as there are another mechanisms of gliadin absorption, i.e. through transcellular pathways. This strategy might therefore be best exploited in combination with other treatments. P(HEMA- co -SS) is another interesting polymer reported to attenuate gliadin-induced changes in permeability and inflammation [11]. Further investigation is required to understand the mechanisms of action and their interaction with human tissues. All these therapeutic candidates are promising, but further studies are required to determine the efficacy, toxicity, and practicality of such treatments. Among these, oral passive antibody has potential as a therapeutic option for CD.

Oral antibody passive immunotherapy is of significant value by virtue of its advantages, including reduced cost, ease of administration, and potential to treat localized conditions in the GIT [17]. Several studies have examined the use of IgY against enteric pathogens such as *Salmonella enteritidis, Salmonella typhimurium* [20], *E. coli O157:H7* [19]*, E. coli 987P* [29], and *Clostridium perfringens* [21]. Antibodies have the ability to bind and sequester gliadin [30, 31], and so we used laying hens to produce IgY specific to gliadin. The use of laying hens as potent antibody producers has numerous advantages over mammalian polyclonal antibody production including the high antibody content, the relative ease of using eggs versus serum, the low cost of animal maintenance, and the availability of large quantities of eggs. Laying hens usually lay about 250 eggs (approximately 4000 g of egg yolk) in a year, compared to the serum collected from a rabbit that is only about 40 ml. One gram of egg yolk laid by the immunized hen contains about 10 mg of IgY whereas 1 ml of rabbit serum yields about 35 mg of IgG [32, 33]. The use of IgY may improve the gliadin-binding efficacy because IgY is polyclonal, and is therefore able to bind to multiple epitopes on various fractions of PT-gliadin. In addition, there is an economical advantage by replacing the expensive monoclonal antibody with inexpensive chicken egg yolk IgY. Our previous *in vivo* mice feeding study has also proved that anti-gliadin IgY prevented > 99 % gliadin absorption in the GIT of mice fed with gliadin and anti-gliadin IgY [16]. Hence, this anti-gliadin IgY produced has potential to be used as a therapeutic oral antibody for CD to neutralize enzyme resistant gliadin in the GIT.

The small-bowel mucosal biopsy organ culture system has been used extensively to clarify the pathogenesis of CD. The advantage of the system comes from the fact that it contains a number of different cell types so that any element of the system can be investigated, from the epithelium through to the mucosal lamina propria. CD researchers have used this model to study the immune mechanisms [4], as well as the contribution of different cytokines [34]. Another investigative method involves biopsy samples. The toxic effect of gliadin is detectable with biopsy samples from active CD patients and short-term treated (less than 3 years on GFD) CD patients who are likely still to have mucosal IgA deposits present. If the CD mucosa is taken from CD patients on GFD for > 4 years, the biopsy samples do not secrete autoantibodies to the supernatant due to overall absence of plasma cells and helper T cells, as well as the failure of memory B cells to become activated [35, 36]. This method has been widely used in studies aiming to clarify the pathogenesis of CD, but it is not widely used in studies related to novel treatment developments. In this study, we employed Caco-2 epithelial cell instead in order to determine the ability of anti-gliadin IgY to inhibit gliadin absorption at epithelial level, as there is no additional benefit to use the model with underlying celiac pathogenesis.

In literature, Caco-2 monolayers have been used to study the inflammatory response induced by gram-positive and -negative bacterial cell surface polysaccharides, teichoic acid, protein A, peptidoglycans, lipid A-associated proteins, lipoproteins and deoxyribonucleic acid, to regulate cytokine synthesis [37]. Peptidoglycans, lipoproteins, lipoteichoic acid, lipopolysaccharide, flagellin and unmethylated CpG dinucleotides in bacterial DNA are also reported to bind Toll-like receptors and induce cytokine synthesis in Caco-2 cells [38, 39].

Caco-2 monolayers is an ideal *ex vivo* model of CD intestinal epithelium because it has been reported to possess a variety of desirable properties upon PT-gliadin stimulation, such as decreased electrical resistance, increased absorption, induced intestinal permeability [12], exerted pro-apoptotic activity [23, 24], and stimulated release of proinflammatory cytokines (such as TNF-α and IL-1β), all upon PT-gliadin stimulation [5]. Caco-2 cell cultures have been employed to study the effects of potential CD treatments, including a zonulin antagonist (a tight junction modulator) [12], Bifidobacteria [5], germinating cereal enzymes [10], and polymeric binders of gliadin [11]. Thus, Caco-2 monolayers are an established model used to evaluate the efficacy of anti-gliadin IgY in neutralizing toxic gliadin at intestinal epithelium level. However, there is also possibility of antibody effect directly on the epithelial cells interfering mechanism of gliadin peptides entrance [40].

Our results show that when the apical chamber was subjected to PT-gliadin, toxic particles flowed from the apical compartment to the basal compartment of the Caco-2 cultures (Table 2). However, the aforementioned flow of peptides was inhibited in the presence of anti-wheat-gliadin and PT-gliadin IgY co-incubated cultures. This is particularly important because it indicates the ability of anti-gliadin IgY to prevent the cytoskeleton rearrangement that promotes the intestinal permeability that is inherent to gliadin exposure. In this context, the minimum ratio of anti-wheat gliadin IgY and PT-gliadin (1:6,000) contributed to maintain the intestinal barrier integrity.

Li and colleagues have reported that there is a dramatic drop of Caco-2 monolayer integrity when the culture medium was changed. This is because glutamine was lost due to a catastrophic loss of electrical resistance. As glutamine is important for intestinal barrier function, it follows that Caco-2 monolayer integrity deteriorated. Membranes were able to recover after 1 h, and it takes up to 2–4 h for protein synthesis to recover. [41]. This is why we incubated PT-gliadin and/or anti-gliadin IgY for 4 h to evaluate their effects on intestinal integrity. Other researchers have reported impaired intestinal integrity of Caco-2 monolayers after 6 h [25], and 3 h [12] exposure to PT-gliadin.

In the present study, PT-gliadin has also shown to trigger the production of pro-inflammatory cytokines (TNF-α and IL-1β), as a result of pro-inflammatory pathways (NF-kB) activation. NF-kB is activated in the small intestinal mucosa of CD patients [42], and gluten peptides have been shown to upregulate the expression of cytokines such as TNF-α [43] and IL-1β [44]. TNF-α detected in the presence of anti-gliadin IgY with intestinal digests of gliadins at a 1:6,000 ratio, demonstrated that anti-gliadin IgY was not enough to neutralize gliadin in the Caco-2 cell cultures. With higher IgY concentration (ratio of 1:3,000), anti-gliadin IgY was able to completely abolish the TNF-α and IL-1β production (Fig. 5). The latter can be explained by inhibition of NF-kB induction due to the gliadin neutralization efficacy of anti-gliadin IgY. NF-kB (subunit proteins: p65 and p50) is made inactive by Ik-B in the cytosol. Exposure to gliadin prompts phosphorylation and results in destruction of Ik-B [45]. After destruction of IkB, NF-kB enters the nucleus and binds with DNA-activating genes that encode for the increased inflammatory mediators (cytokines) production, leading to cellular dysfunction and tissue destruction [46].

TNF-α together with IL-1β are the important cytokines that are involved in NOS activation, both of which act as mediators to facilitate the interaction of intraepithelial lymphocytes and intestinal epithelial cells promoting tissue inflammation [47]. In addition, TNF-α also has a positive effect on attraction of neutrophils, which cause perpetuation of inflammatory responses, cell damage, and eventually epithelial barrier dysfunction [5].

There are several attempts reported in literature on treatment of CD by inhibiting gliadin-induced inflammatory responses at intracellular or submucosal level, such as zonulin antagonist [12], tissue transglutaminase inhibitors [13], peptide vaccine [14], and NKG2D/MICA blocker [15].

In our previous study we used anti-gliadin IgY antibody to show its inherent ability in a diagnostic role. With anti-gliadin IgY used as capture antibody and biotinylated mAb as detecting antibody, a highly sensitive DAS-ELISA was developed, with linear standard range of 4–40 ng/ml and the limit of detection equivalent to 0.8 ppm in foods [25]. In the present study we have used anti-gliadin IgY antibody to establish its potential therapeutic role. The anti-gliadin IgY antibody used in this study aims to neutralize intestinal digests of gliadin peptides (PT-gliadin), inhibiting gliadin-induced cytotoxic and pro-inflammatory responses at intra-luminal level before they enter the small intestine, ultimately preventing the gliadin induced inflammatory events in CD. Thus, oral administration of anti-gliadin IgY may contribute to maintaining healthy and normal intestine by masking ingested gliadin peptides. However, it warrants further *in vivo* animal and human studies for anti-gliadin IgY to reach its realm of CD therapeutics.

## Conclusion

In summary, the anti-wheat gliadin IgY antibody produced in this study has proved to inhibit the absorption of gliadin and release of gliadin-induced inflammatory response in Caco2 cell culture model of CD. As a result, the epithelial monolayer of the small intestine remains undisturbed upon gliadin stimulation. The reported data extends the spectrum of functional effects attributable to IgY antibodies, and may provide a rationale for their potential role in other diseases where gastrointestinal immune function is triggered by antigens other than gliadin.

## Methods

### Pepsin-trypsin-gliadin (pt-gliadin) preparation

A pepsin and trypsin resistant gliadin (PT-gliadin) was prepared according to the method previously described with minor modifications [12]. Each 50 g Sigma gliadin (G-3375; Sigma, St. Louis, MO) or 50 g wheat flour was dissolved in 500 ml 0.2 N HCl for 2 h at 37 °C with 1 g pepsin (P-7000; 800–2,500 units/mg protein, Sigma, St. Louis, MO). The resultant peptic digest was further digested by addition of 1 g trypsin (P-8096, activity, 4x USP specifications; Sigma, St. Louis, MO), after pH adjusted to 7.4 using 2 M NaOH. The solution was stirred vigorously at 37 °C for 4 h, boiled to inactivate enzymes for 30 min, lyophilized, and then stored at −20 °C until used. PT-gliadin was freshly suspended in a sterile phosphate buffered saline (PBS, 0.15 M NaCl, 0.0027 M KCl, 0.0081 M disodium phosphate and 0.0015 M monopotassium phosphate, pH 7.2) to a final concentration of 1 mg/ml. PD-casein (BactoTryptone, Sparks, MD) was used as a negative control in the experiment.

### Production of IgY antibody

Laying hens were cared for in accordance with the guidelines of animal warfare of the Canadian Council on Animal Care (CCAC 2000), approved by University of Alberta Animal Care and Use Committee (protocol AUP00000163). Immunization of hens was carried out as previously described [31]. Each Sigma gliadin, wheat gliadin, or PT-gliadin (500 μg of protein/ml) was suspended in PBS (pH 7.2) and emulsified with an equal volume of Freund’s Incomplete Adjuvant (Sigma, St. Louis, MO). Eighteen 23-weeks-old Single Comb White Leghorn chickens were subcutaneously injected with each emulsion of Sigma gliadin (*n* = 6), wheat gliadin (*n* = 6) or PT-gliadin (*n* = 6). Booster immunizations (500 μg of protein/ml) were given after 2 and 6 weeks of the initial immunization. Eggs were collected daily and stored at 4 °C until the extraction of the antibodies.

### Purification of IgY antibody

The egg yolks from hyperimmunized hens were physically separated from the egg white and first mixed gently with eight volumes of cold distilled water (acidified with 0.1 M HCl to give pH 4.0) to avoid possible disruptions of egg yolk granules due to the presence of high concentrations of acid. Cold acidified distilled water (pH 2.0) was then added to make a final dilution of 1:10. After mixing well, the mixture was adjusted to a pH 5.0-5.2 and incubated at 4 °C for 12 h. The WSF was obtained by centrifugation at 3.125 x g at 4 °C for 20 min. The supernatant was collected as the IgY rich WSF and titrated by indirect ELISA (mentioned below) using Sigma gliadin as a coating antigen. The WSF (10 mg protein/ml) of high titre was further purified by using a 1.0 x 110 cm column of Sephacryl S-300 (GE Healthcare, Piscataway, NJ) which was equilibrated and eluted with PBS at a flow rate of 3 ml/h. Blue dextran (Pharmacia Biotech Inc., Baie-d’Urfe, QC) and titrated water were used to determine void volume (V_o_) and total volume (V_t_) of the column, respectively. The partition coefficient was calculated from the formula: K_av_ = (V_e_ - V_o_)/(V_t_ - V_o_), in which V_e_ represents the volume of the peak fraction. The eluates (1 ml) were analyzed for IgY activity at 405 nm by ELISA. The eluates of IgY were pooled, freeze-dried and analyzed for protein content, total IgY and specific IgY.

### Quantitative ELISA for anti-gliadin IgY

Unless indicated otherwise, all incubations were performed at 37 °C with four washes of PBS-T each step. Microtiter plates were coated with 100 μl of rabbit anti-chicken IgG (final concentration of 2 μg per well) or gliadin (10 mg/ml of 60 % ethanol) for 1 h. Non-specific binding sites were blocked with 120 μl of 3 % BSA solution (W/V) in PBS-T for 45 min. To each well, 100 μl of of purified chicken IgG standard, WSF (diluted 1:1,000 in PBS-T) or column fraction (diluted 1:3,000 in PBS-T) was added as a specific IgY, and non-immunized IgY prior to incubating for 1 h. Two-fold serial dilutions of purified chicken IgG in PBS (0.5 to 0.031 μg/ml) were used as a reference antibody to prepare a standard curve on the same plate.

Plates were subsequently added with 100 μl of rabbit anti-chicken IgY conjugated with HRP (diluted 1:5,000 in PBS-T) and incubated for 90 min. After washing, 100 μl of freshly prepared substrate solution, 2,2′-Azinobis (3-ethylbenzothiazoline-6-sulfonic acid)-diammonium salt in 0.05 M phosphate citrate buffer (pH 5.0) containing 30 % hydrogen peroxide was added. OD 405 nm was taken after 30 min using an ELISA Vmax kinetic microplate reader. The ELISA value of antibody activity was determined by subtracting the value of the control antibody from that of specific antibody.

### Preparation of Caco-2 cell culture system

Caco-2 cell culture (ATCC, Rockville, MD) was performed as previously described [48]. Caco-2 cells (passages 20–24) were maintained at 37 °C in (DMEM) complemented with 4 mM glutamine, 100 U/ml penicillin, 100 U/ml streptomycin, 1 % non-essential amino acids, 10 % heat inactivated fetal calf serum, in an atmosphere of 90 % air and 10 % CO_2_. Cells were cultured on 24-well culture plates with polyethylene terephthalate membrane inserts (pore size 1.0 μm; BD Bioscience, Mississauga, ON) at a density of 3 × 10^5^ cell/cm^2^. The medium was changed three times a week and maintained for 21 days in medium for complete differentiation.

The integrity and permeability of the monolayer was determined by measuring the TEER value using Epithelial voltohmmeter (World Precision Instrument; Haven, CT) according to manufacturer’s instructions. The final values were expressed as Ω × cm^2^ on the basis of the following equation: TEER = (R - Rb) × A, where R is the resistance of filter insert with cells, Rb is the resistance of the filter alone and A is the growth area of the filter in cm^2^. The transepithelial electrical resistance values obtained in the absence of cells were considered as background measurements. All experiments were started based on the TEER of the monolayer when it reached 300–400 Ω cm^2^ at 21 days after seeding cells on the transwell inserts.

In all experiments, phenol red was included in the apical chamber to estimate paracellular diffusion through the Caco-2 cell monolayer. The percentage of phenol red transported into the basal chamber was calculated as previously described [49]. Briefly, 500 μM phenol red was subjected to the apical chamber. Aliquots of 100 μl were removed from the basal chamber after 2 h incubation at 37 °C and added with 1 M NaOH. The OD of the basal chamber contents was measured at 558 nm to detect any leakage of the phenol red through the intercellular spaces.

### Neutralization experiments of IgY against PT-gliadin

Caco-2 cell culture was used to determine the PT-gliadin neutralization effect of anti-gliadin IgY. After formation of Caco-2 monolayer, the 24-well Caco-2 culture medium in the basal chamber was removed and replaced with 1.2 ml phenol red free HBSS. Caco-2 cells on the transwell inserts were also washed with phenol red free HBSS and replaced with 300 μl phenol red free HBSS. One hundred μl of 7 different experimental conditions were added to the wells (*n* = 3): A) Control: PBS; B) Negative control: PD-casein (60 μg); C) PT-gliadin (60 μg); D) Non-specific IgY (40 ng); E) anti-gliadin IgY (10 ng) and PT-gliadin (60 μg) [1:6,000]; F) anti-gliadin IgY (20 ng) and PT-gliadin (60 μg) [1:3,000]; G) anti-gliadin IgY (40 ng) and PT-gliadin (60 μg) [1:1,500]. This experiment was repeated 5 times in different plates. After 1 h, 2 h and 4 h incubation, the basal media were recovered, and quantified for diffusion of phenol red and PT-gliadin content by measuring the absorbance at 558 nm and ELISA, respectively. After each experiment, the TEER values were measured in all inserts to determine the effect on the intestinal integrity of each experimental condition (A-G).

### Quantification of gliadin

A commercially available quantitative immune-based ELISA kit (Crystal Chem, Inc., Downers Grove, IL) was used to quantify PT-gliadin in the apical and basal media of the Caco-2 monolayers according to the manufacturer’s instructions. The analyses were performed in the apical and basal media after 1, 2 and 4 h of incubation.

### Quantification of inflammatory markers

Caco-2 cells (passages 20–24) were seeded at 50,000 cell/cm^2^ on 6-well plates and grown with DMEM; culture media were changed every 2 days under the condition as mentioned above. Experiments were performed 5 days post-seeding. PT-gliadin and/or anti-gliadin IgY was exposed to cells for 24 h. Supernatants of Caco-2 cells cultures of each experimental condition was used to quantify TNF-α (Cat no: 88–7346; eBioscience, San Diego, CA) and human IL-1β (Cat no: 88–7010; eBioscience, San Diego, CA) by ELISA system according to the instruction of the manufacturers with a linear range of 4–500 pg/ml. The results of these assays were expressed as pg/ml of media. The experiments were performed in triplicate for three times.

### Statistical analysis

The data are presented as the arithmetic mean for each experimental point ± SD. For anti-gliadin IgY production, student *t*-test (one-tailed *t*-test) was used to analyze for significant differences (*P* < 0.05) between the control (non-immunized) and samples. For cell culture study, statistical calculations were performed using ANOVA. Differences among 7 conditions were examined using a Turkey post hoc test. Statistical significance at p value of 0.05 or less was considered significant.

## Abbreviations

CD: Celiac disease
PT-gliadin: Pepsin and trypsin resistant gliadin
IgA: Immunoglobulin A
IL-1β: Interleukin 1 beta
TNF-α: Tumor necrosis factor alpha
GFD: Gluten free diet
IgY: Immunoglobulin Y
PD: Pancreatic digested
PBS: Phosphate buffered saline
WSF: Water soluble fraction
ELISA: Enzyme-linked immunosorbent assay
PBS-T: PBS-Tween
BSA: Bovine serum albumin
HRP: Horse radish peroxidase
OD: Optical density
DMEM: Dulbeco’s Modified Eagle’s Medium
TEER: Transepithelial electrical resistance
HBSS: Hank’s Balanced Salt Solution
ANOVA: One-way analysis of variance
GIT: Gastrointestinal tract
